# Supplementary material for: DMRforPairs: identifying Differentially Methylated Regions between unique samples using array based methylation profiles
Source: BMC Bioinformatics. 2014 May 15;15:141. doi: 10.1186/1471-2105-15-141 (PMC4046028; doi:10.1186/1471-2105-15-141)
Supplement: Additional file 2 — DMRforPairs output for the comparison of A431-MCF7 and NA17018-NA17105. Please start from the HTML files in each folder. Available via the BMC Bioinformatics website. [file 1471-2105-15-141-S2.zip › 1394847754114233_MOESM2_ESM/A431_MCF7/figures/34228.pdf]

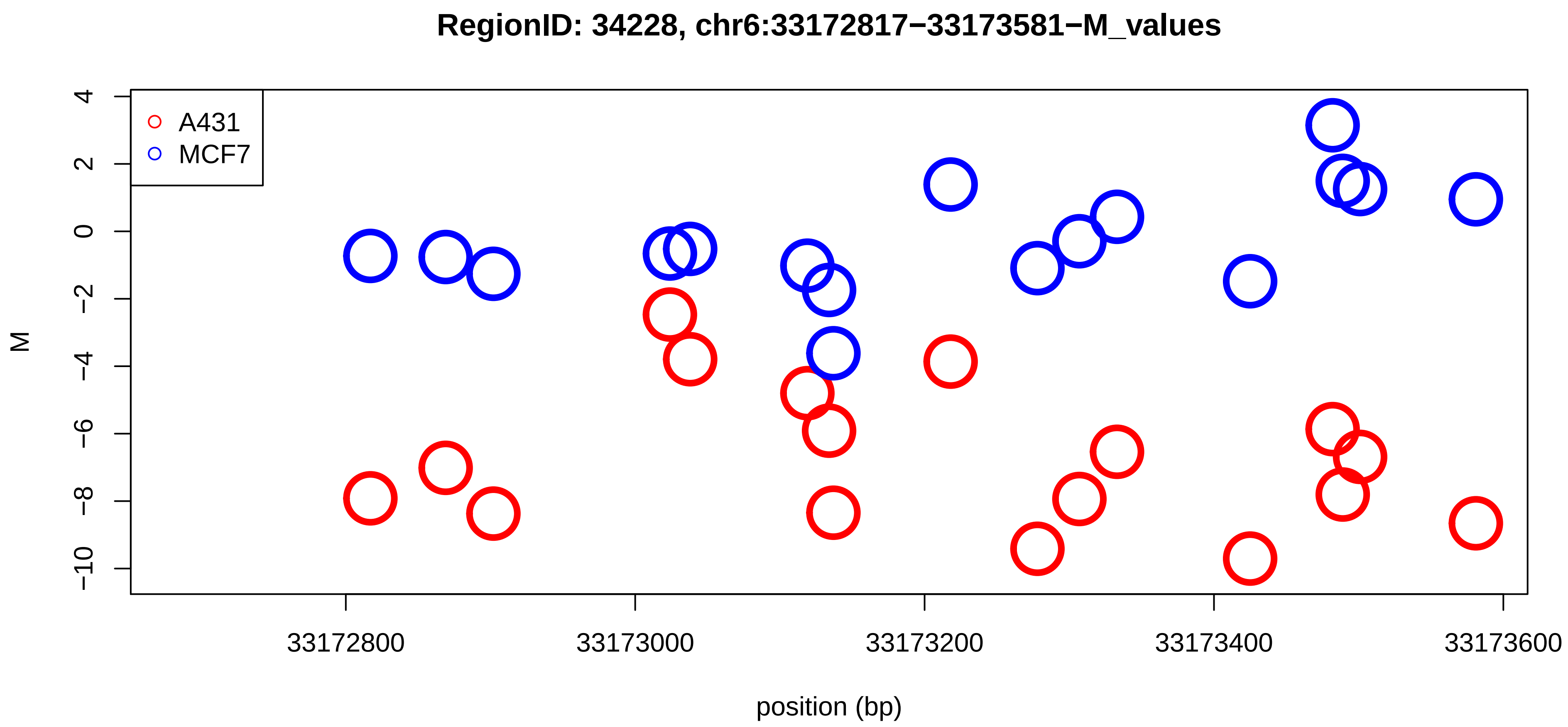

RegionID: 34228, chr6:33172817−33173581−M_values
A431
MCF7
M
4
2
0
−2
−4
−6
−8
−10
33172800
33173000
33173200
33173400
33173600
position (bp)

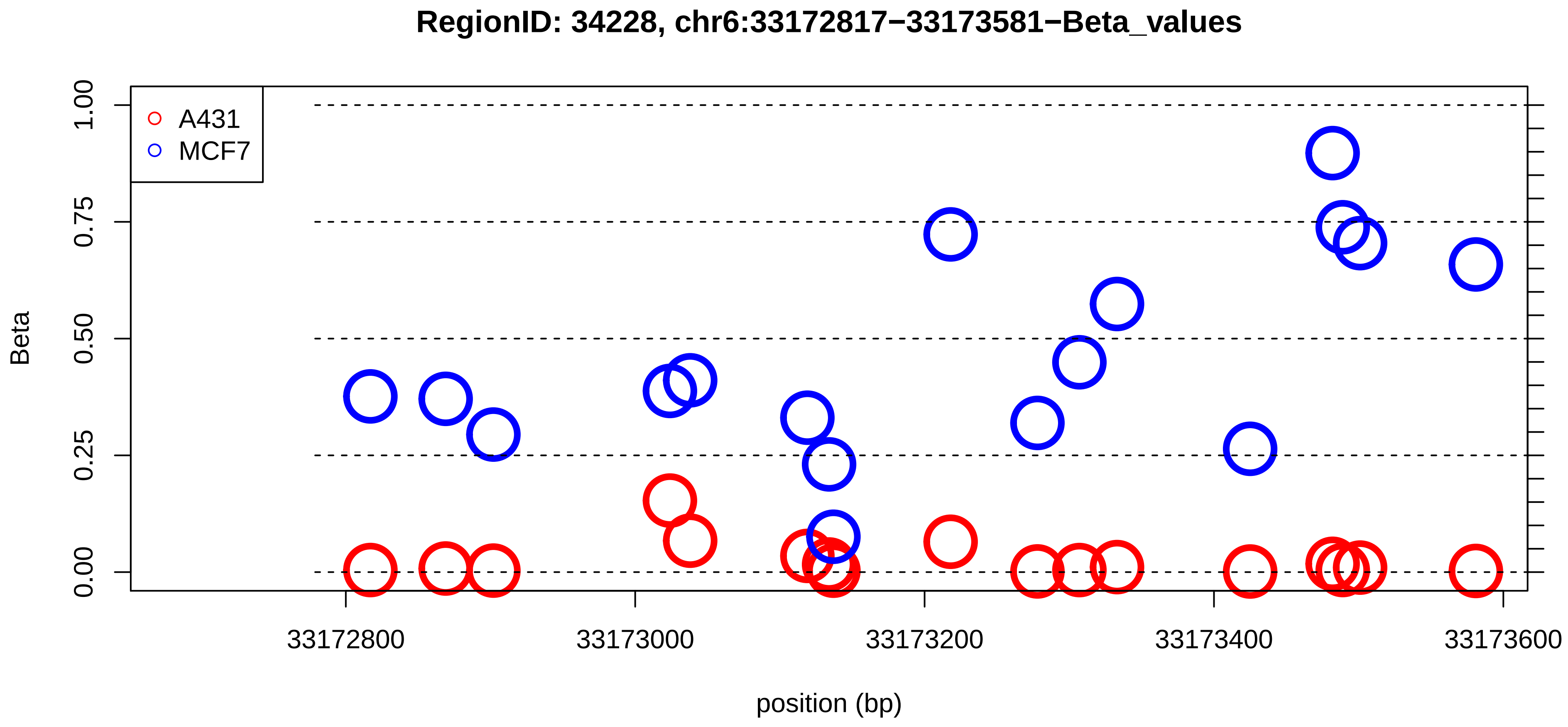

RegionID: 34228, chr6:33172817−33173581−Beta_values
A431
MCF7
Beta
1.00
0.75
0.50
0.25
0.00
33172800
33173000
33173200
33173400
33173600
position (bp)

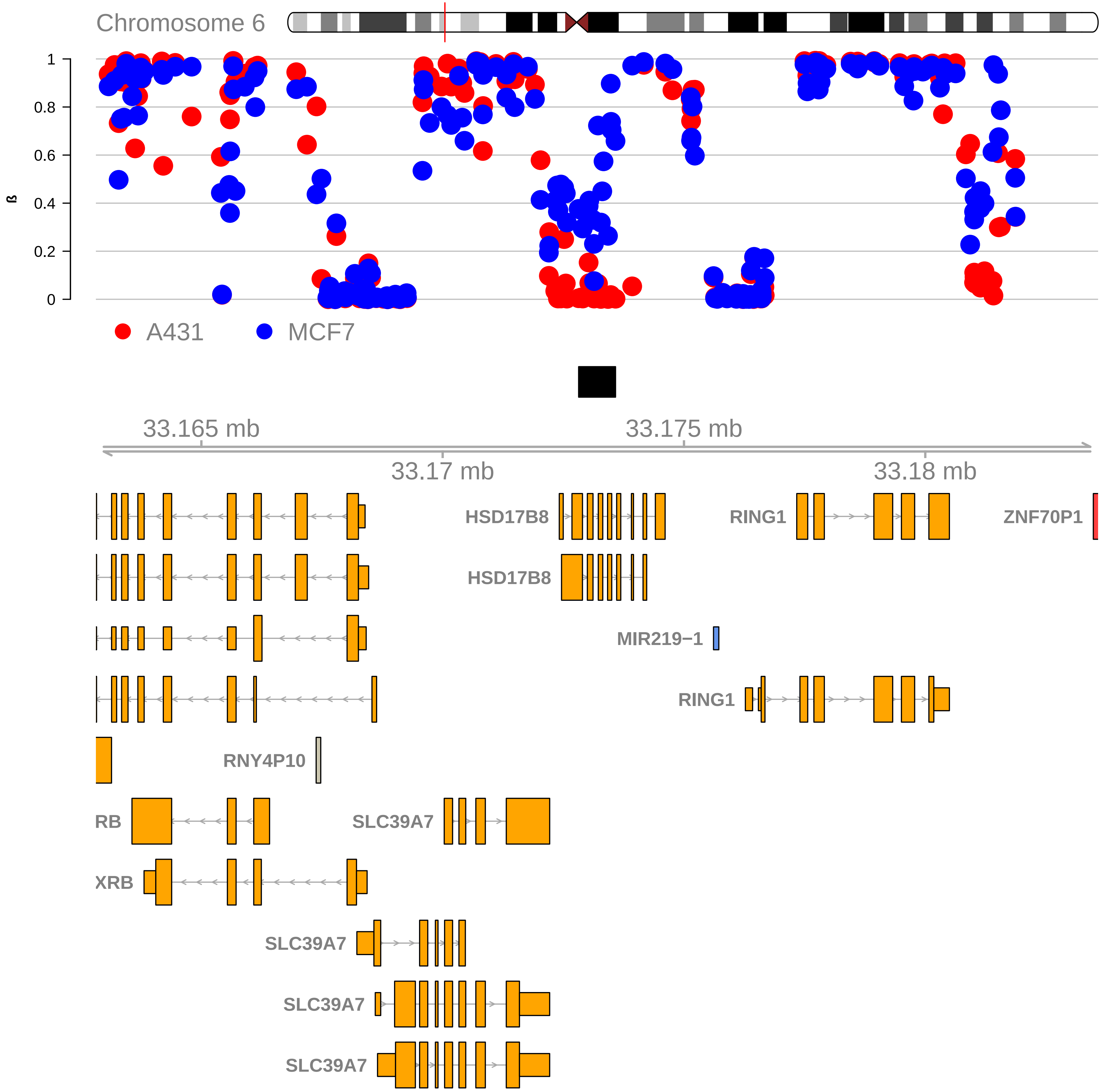

Chromosome 6
β
1
0.8
0.6
0.4
0.2
0
A431
MCF7
33.165 mb
33.175 mb
33.17 mb
33.18 mb
HSD17B8
RING1
ZNF70P1
HSD17B8
MIR219−1
RING1
RNY4P10
RB
SLC39A7
XRB
SLC39A7
SLC39A7
SLC39A7
